# Great expectations: Aligning visual prosthetic development with implantee needs

**DOI:** 10.1101/2024.03.12.24304186

**Published:** 2024-03-14

**Authors:** Lucas Gil Nadolskis, Lily Marie Turkstra, Ebenezer Larnyo, Michael Beyeler

**Affiliations:** 1 Interdepartmental Graduate Program in Dynamical Neuroscience, University of California, Santa Barbara; 2 Department of Psychological & Brain Sciences, University of California, Santa Barbara; 3 Center for Black Studies Research, University of California, Santa Barbara; 4 Department of Computer Science, University of California, Santa Barbara

## Abstract

**Purpose:**

Visual prosthetics have emerged as a promising assistive technology for individuals with vision loss, yet research often overlooks the human aspects of this technology. While previous studies have concentrated on the perceptual experiences of implant recipients (implantees) or the attitudes of potential implantees towards near-future implants, a systematic account of how current implants are being used in everyday life is still lacking.

**Methods:**

We interviewed six recipients of the most widely used visual implants (Argus II and Orion) and six leading researchers in the field. Through thematic and statistical analyses, we explored the daily usage of these implants by implantees and compared their responses to the expectations of researchers. We also sought implantees’ input on desired features for future versions, aiming to inform the development of the next generation of implants.

**Results:**

Although implants are designed to facilitate various daily activities, we found that implantees use them less frequently than researchers expected. This discrepancy primarily stems from issues with usability and reliability, with implantees finding alternative methods to accomplish tasks, reducing the need to rely on the implant. For future implants, implantees emphasized the desire for improved vision, smart integration, and increased independence.

**Conclusions:**

Our study reveals a significant gap between researcher expectations and implantee experiences with visual prostheses, underscoring the importance of focusing future research on usability and real-world application.

**Translational relevance:**

This work advocates for a better alignment between technology development and implantee needs to enhance clinical relevance and practical utility of visual prosthetics.

## Introduction

Visual neuroprostheses, such as retinal and cortical implants (colloquially referred to as “bionic eyes”), have emerged as a promising assistive technology for individuals with blindness^1–7^. Analogous to cochlear implants, these devices electrically stimulate surviving neurons in the visual pathway to evoke visual percepts (phosphenes)^8,9^. Existing devices generally provide an improved ability to localize high-contrast objects and perform basic orientation & mobility tasks^4,10^. Notable examples include Argus II^1^ (Second Sight Medical Products, Inc., Sylmar, CA), the first retinal implant to obtain FDA approval, and its successor, Orion^7^ (Cortigent, Inc., Valencia, CA; formerly Second Sight), a cortical implant that is currently in clinical trials (clinicaltrials.gov: NCT03344848). Whereas Argus II has been implanted in 388 Argus II recipients worldwide (157 female and 231 male; personal communication with Cortigent, Inc, 2024.), Orion started its clinical trial in the US with six implant recipients (1 female and 5 male), of which three remain implanted.

Deeply rooted in the field of ophthalmological restorative medicine, most current research in this field adheres to the medical model of disability^11^; that is, a perspective which sees blindness as a result of an individual’s physical impairment that can be “fixed” – in this case, with an invasive prosthesis. As pointed out by Refs.^12–14^, the majority of research on visual prostheses (and more generally: low vision aids) has primarily focused on technological and functional aspects of these implants (e.g.., the ability to produce phosphenes and the resulting Snellen acuity) and has rarely incorporated implant recipients (*implantees*) in the decision-making and design process^14^. However, blindness is not just about one’s physical impairment, but also about the individual’s subjective psychological experience and the societal contexts in which they live^15,16^. In the development and evaluation of assistive technologies for people who are blind, it is crucial to focus not only on the technical aspects but also on the wants, needs, and lived experiences of the end users, studying how they might utilize such devices within their daily lives. This approach ensures that technology serves the user, enhancing their quality of life rather than solely aiming to correct a physical condition.

Although tools and surveys have been developed to provide a subjective assessment to capture the functional visual ability and well-being of an implantee^10,17,18^, in practice these are often employed as an external validation tool that constitutes the very last step of the design process^44,17^. It is therefore perhaps not surprising that none of the current devices have found broad adoption, and that several device manufacturers had to close their doors because their device did not (such as in the case of Retina Implant AG) lead to “the concrete benefit in everyday life of those affected”^1^.

This lack of end-user involvement and limited adoption underscores the necessity for a deeper exploration into how implants are actually used in daily life, contrasted with the initial expectations of their designers. Despite numerous studies assessing functional vision^4,6,17^ and documenting the experiences of current implantees^10,13,19,20^, as well as discussions on ethical considerations in trial participant selection^21,22^ and the attitudes of blind individuals toward implant technology^23^, a comprehensive understanding of the real-world application of these devices remains elusive.

This study aims to explore the perspectives, experiences, practices, and aspirations of individuals who have received one of the most commonly available visual implants (Argus II or Orion). It also seeks to contrast these user insights with the viewpoints of prominent researchers who are either involved in developing these devices or who interact directly with the implantees. We also sought feedback from implantees to identify current technology limitations and gather suggestions for future enhancements. Through reconciling the viewpoints of both researchers and implantees as well as fostering cooperative efforts in the design process, we hope that the next generation of visual prosthetic technology can have a profound impact on the quality of life of millions of people worldwide.

## Methods

We conducted semi-structured interviews with 12 participants (six researchers and six implantees) to assess the actual frequency of implant usage among Argus II and Orion users, and compared the reported usage to researcher expectations. This sample represents roughly 1% of the global Argus II population and 67% of the individuals who still have the Orion implant, reflecting the rarity of these implants in both commercial and clinical settings. We initially posed structured questions covering an extensive array of instrumental activities of daily living^22–25^ (iADLs). We then engaged in open-ended discussions to delve into which strategies and usage patterns that implantees and researchers deemed effective or ineffective, and what the implantees hope to see in the next generation of implants.

Informed consent was obtained from all participants after confirmed understanding of the expectations of the interview. The study was deemed exempt from review by the Institutional Review Board of the University of California, Santa Barbara.

### Study participants

Twelve participants (2 female and 10 male) were recruited via email and phone through a combination of snowball sampling and connections with various research groups and previous collaborators (Tables 1–2). To qualify for the study, implantees (I1–6) had to be current recipients of either the Argus II or Orion implant. All implantees have had their implant for at least five years, remain currently implanted with their respective devices, and none had reported medical complications with the device. Five out of six implantees lived with either family or a spouse while one lived alone, and all were frequent users of assistive technology both inside and outside of the home. In addition, four out of five reported to be users of either a cane or a guide dog, while one reported using both and one preferred not to answer. All participants resided in either the United States or the Netherlands.

The other six participants (R1–6) were researchers, either principal investigators or medical professionals, who are currently working with Orion, or had previously worked with either Orion or Argus II within the last five years. R2, R3, and R6 had experience with both Argus II and Orion. None of the researchers reported having any visual impairment.

### Interview procedure

The interviews were conducted via Zoom by the two lead researchers of the study, one blind and one sighted. Transcripts were generated using the Otter AI transcription software (Mountain View, CA) and analyzed manually by the research team. Each interview lasted between 30 and 90 minutes.

Three probing interview questions were presented to implantees in order to further understand their experience with their device:

- Q1-I: How often do you use your implant for <iADL>? Choose from: daily, weekly, monthly, yearly, never.
- Q2-I: Please give some examples of how your implant supports <iADL>. What works? What does not?
- Q3-I: What do you wish your implant could do to support/facilitate <iADL>?

These questions were repeated for each of twelve iADLs, which we drew from previous literature^24–27^ to ensure a broad spectrum of everyday tasks. These iADLs included essential activities such as meal preparation, housekeeping, transportation, and socializing, chosen for their relevance to independence and quality of life for blind individuals. For each of these iADLs, we formulated a series of questions aimed at uncovering not only the frequency and extent of visual prosthetic use, but also the practical benefits and limitations experienced by implantees.

In addition, we sought to understand the discrepancy between the expected and actual use of these devices. Researchers were therefore presented with a similar set of questions, but were asked to reply based on their perception of an implantee’s device usage:

- Q1-R: How often do you expect implantees to use the implant you currently work with for <iADL>? Choose from: daily, weekly, monthly, yearly, never.
- Q2-R: Please give some examples of how the implant you currently work with might support <iADL>.

### Data analysis

To gain a quantitative understanding of device usage, we converted the answers to Q1-I and Q1-R to a numerical five-point scale (0: never, 1: yearly, 2: monthly, 3: weekly, 4: daily), and used a linear mixed model to test whether the answers of implantees and researchers were statistically different. This is a valid transformation, since the original data is ordinal in nature (i.e., “daily” > “weekly”, “weekly” > “monthly”) and our mapping to numerical values preserves this order. The linear mixed model used group (“researcher” vs. “implantee”) as a fixed effect and random intercepts for each subject to account for individual differences. The analysis included 144 observations across 12 unique subjects, with each subject contributing 12 observations. The model was estimated using restricted maximum likelihood (REML) via the <monospace>statsmodels</monospace> (v0.14.1) package in Python 3.10. We further performed pairwise Tukey’s Honest Significant Difference (HSD) tests for each iADL to test the hypothesis that implantees used their device less frequently than researchers expected.

To get a qualitative understanding of the device usage for the different iADLs, we performed an inductive thematic analysis^28^ on Q2-I, Q-R, and Q3-I. Themes were individually identified from transcripts, with new ones added for unclassified examples. This iterative process continued across all transcripts until no new themes emerged, culminating in a consensus on 13 definitive themes. Both implantees’ and researchers’ responses were categorized under these themes, with unique codes assigned to each for systematic analysis.

## Results

### Implant usage expectations vs. reported outcomes

The responses to Q1-I and Q1-R for each iADL are shown in Table 3. Participant I3 was reportedly the most prolific implant user, relying daily on his Orion device for several indoor and outdoor activities. He particularly values the device’s assistance outdoors where it complements his mobility cane and aids him in navigating streets, identifying buildings, and discerning the status of doors, notably on buses and trains. Participant I1 reported using his implant occasionally for laundry and socializing (“monthly”) and rarely for housekeeping, shopping, or hobbies (“yearly”). Participants I2 and I5 both use their implants for transportation (with limited success) and in social settings.

However, the most common answer to how often implantees used their device for a certain iADL was: “never.” I4 and I6 reported that they never used their implant for any everyday activities. Though some may have made use of their implants for these activities in the past, none of the implantees reported currently using their implant for meal preparation, reading, managing finances, managing medication, or using personal electronic devices.

This is in stark contrast to the responses of the interviewed researchers, who expected the implant to apply to all iADLs at least to some degree. R3, R5, and R6, who had more device-centric roles at their jobs, expected the implant to be used daily or weekly for most iADLs, although R6 acknowledged that actual device usage may vary from person to person. All researchers agreed that socialization would likely be the most prolific use of the implant. R5 was especially adamant about his expectation of the implant being useful for every iADL on the list except for employment.

**Figure 1:**
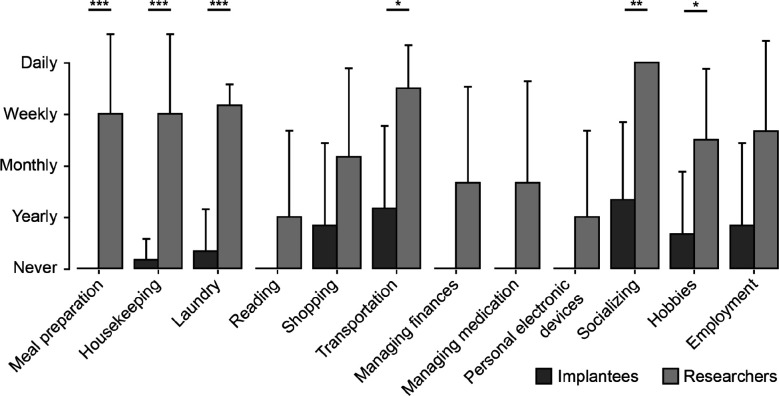
Implantee and research perceptions implant use frequency for each iADL. Same data as presented in Table 2, converted to a numerical five-point scale (0: never, 1: yearly, 2: monthly, 3: weekly, 4: daily). Significant differences between implantee and research perceptions (corrected for multiple comparisons using Tukey’s Honest Significant Difference test) are denoted as *: p<.05, **: p<.01, ***: p<.001.

To allow for a more quantitative comparison between researcher and implantee responses, we converted these answers to a numerical five-point scale (Figure 1) and used a linear mixed model to confirm that the answers of implantees and researchers were statistically different. The group effect (researchers vs. implantees) showed a significant difference in rating values (*z* = 4.723, *p* < 0.001), indicating a strong influence of group membership on the outcome. Specifically, researchers rated the frequency of device usage significantly higher than implantees, with an estimated increase of 2.0 in rating value (e.g., from “yearly” to “weekly”, or from “monthly” to “daily”) as compared to implantees (SE = 0.423, *z* = 4.723, *p* < .001, 95% CI [1.170, 2.830]). The variance attributed to random intercepts for subjects was estimated to be 0.407, suggesting variability in rating values across subjects. The model’s convergence was successfully achieved, ensuring the reliability of these estimates.

In pairwise comparisons using Tukey’s Honest Significant Difference (HSD) test, researchers were consistently found to have significantly higher frequency ratings compared to implantees (*p* < .05) for meal preparation, housekeeping, laundry, transportation, socializing, and hobbies, with mean differences ranging from 1.8333 to 3.0.

### Examples of implant usage in the daily life of implantees

To gain insight into the everyday use of their devices by implantees, we solicited specific examples (Q2-I and Q2-R) and compared these with researchers’ expectations of how their devices would be used in implantees’ daily life. Activities were sorted by their reported frequency of occurrence, as gathered from our interviews, and the most commonly performed activities are discussed below.

#### Transportation

One application area that both implantees and researchers agree to be of potential value for a visual implant user is that of transportation. Implantees who live in urban areas remarked that the device proved useful for stepping in and out of the bus, avoiding bumping into walls and other obstacles, and for detecting people when entering and exiting a train. However, the implant could rarely replace the use of a mobility cane or a guide dog completely. In the words of Participant I4:

“I use my device in combination with my guide dog - he walks me to the front of a shop. [Then I use the implant] mostly for orienting myself inside.”

In contrast, Participants I1 and I6, who reside in the city, report that they never use the implant for navigation. Further inquiry revealed that their limited use did not stem from a lack of effort. Specifically, I6 noted that the ideal scenario for using the implant would be assistance with street-crossing. Yet, the implant’s artificial vision proved too inundating and lacked the necessary detail for her to feel secure using it for navigation in a busy city setting, leading her to prefer being driven as a more reliable alternative. She recounts:

“I remember my experience crossing the street with the Argus, and that I decided to turn off the stimulation because there were too many flashing lights. The ideal way for me to cross the street is to be able to focus on the important points and detect the distance when crossing…without so much stimulation.”

Participants I1, I2, and I5 were quick to remark that the implant slowed them down, as they had learned to (e.g.) navigate much faster and more reliably with a mobility cane. Participant I2 hoped to use the implant for navigation, but remarked that it did not provide any concrete benefit:

“I have kind of given up on using it outdoors [...], because I hoped it would serve as a navigation device, or something to help me get my bearings and aid in mobility. But it really doesn’t bring any benefit. Lately, I’ve been involved in research studies, just trying to help people understand and advance the science around it, but it doesn’t really provide anything useful enough for me to use in my daily life at all. I don’t know if anybody really does [use it in daily life] at this point.”

#### Socializing

Participant I3 found the implant extremely useful for orientation in social environments, enabling him to monitor the movements of people arriving and departing. This feature was especially helpful for recognizing when someone was approaching to engage in conversation, departing, or coming back, thus helping I3 discern the presence of others nearby. I2 agreed:

“You know, if you’re sitting at a table, you could maybe tell if somebody was getting up and walking away. Sometimes people have gotten up and walked away while you’re talking, and you end up talking to an empty table. So, there could be some minimal benefit to having the [implant] in a social or entertainment environment.”

Participant I1 emphasized the implant’s significant role in enhancing social experiences and memorable moments, stating:

“I use the implant everytime that something new happens to see what I can see. So far I have used it for seeing my birthday candles, fireworks and going to baseball games.”

All researchers similarly emphasized the importance of socialization, and how the implants might facilitate this instrumental aspect of daily life. Various use cases of the implant in socialization settings were mentioned, with R1 providing some specific context as to how the implant might have aided in a situation similar to that of I2 above:

“When people use their implants … they can perceive movements: if someone just left, or if the flash of the person that was right in front of them is not there anymore - they can actually pick that up. [The person in front of them] could be anybody, and they won’t be able to recognize them as their friend or anything, but at least it’s some information.”

#### Meal preparation

None of the implantees reported using their devices for meal preparation, although most researchers believed the implant could be somewhat helpful. Implantees mentioned that living with family or a spouse, along with utilizing other assistive tools already in place for cooking, proved more useful for these tasks.

R1 provided insights into how the device could potentially complement other assistive tools in meal preparation if an implantee chose to use it for this purpose:

“One aspect of the process for individuals who’ve undergone blind rehabilitation involves having an occupational therapist visit their home to label items and make modifications for easier navigation. This can include adding high-contrast colors to cabinets or using tape and paint for visual cues. With these adaptations in place, introducing an implant can further assist by enhancing their ability to perceive contrasts, helping them locate items by size, and distinguish between things like salt and pepper during meal preparation. Although these improvements might seem basic, they could significantly ease daily activities.”

Other researchers expressed skepticism regarding the device’s suitability for meal preparation, offering more cautious perspectives on its effectiveness as a standalone aid. R6 elaborated on these views:

“The device performs much better when you’re in high contrast situations, and a kitchen is not necessarily a high contrast situation. So I would anticipate that this is not the ideal usage situation for a system like this.”

Implantees reported not currently using their devices for meal preparation tasks, primarily due to safety concerns, reliance on existing strategies, and assistance from other tools and cohabitants. I1 and I4 highlighted a lack of specific training on utilizing the implant in the kitchen, leading them to prefer established routines. I1 encapsulated their perspective, stating:

“I don’t really think I’ve had any success using the device in the kitchen. I can’t even come up with any use cases at the moment.”

#### Housekeeping, laundry, and tidying

Regarding housekeeping activities like laundry and maintaining general organization, several implantees noted their attempts to incorporate their implants, albeit with challenges. I5 had previously tried to use his implant to do laundry, including sorting his socks, but found that:

“The glasses would actually be more of a problem than a solution - and the cord would get in the way.”

Other implantees found more success when applying their implants to similar tasks. They noted the implant’s utility for specific housekeeping duties, with I1 highlighting the ability to discern whether lights were on or off, aligning with the device’s effectiveness in high-contrast situations.

Furthermore, researchers had expectations for the implants to facilitate housekeeping activities, especially once an implantee’s home had been customized to suit their needs. R1 commented:

“Assuming that their home is already modified to help them with these things… I would think that having an implant would only enhance housekeeping, it can actually enhance the contrast or things of that nature for the objects that they are looking at.”

#### Reading

While current implants fall short of enabling the reading of fine print and text, R6 challenges the notion of prioritizing reading capabilities in future implant developments, pointing to the superior utility of existing assistive technologies like audiobooks and screen readers. R6 stated:

“I think text to text-to-voice systems are so advanced that it doesn’t make sense to use the device to read. I see no reason why anybody needs to use the device to read anymore, other than for the pleasure [or joy] of being able to read letters.”

Conversely, R5 highlighted the desire among potential future implantees to regain some form of reading ability, noting the varied proficiency levels among blind and low-vision individuals with braille and screen readers, and the general wish to read again. Echoing this sentiment, I1 expressed a specific desire:

“I would love to be able to read stop signs or road signs. Just having these signs been read to me in some capacity would be great.”

#### Other activities of daily living

Despite not being presented as an iADL in our interviews, one common theme between implantees and researchers was the mention of using the device to locate lost, dropped, or missing objects. Participant I1 specifically mentioned using their implant more frequently to locate their smartphones and computers than to actually operate these personal electronic devices.

However, Participant I3 (the most prolific implant user in our sample) found less use for the implant for activities that require navigating websites and reading, such as managing his finances. He summarized his thoughts as follows:

“The reason I no longer use my implant in these different daily activities is because it doesn’t provide a real benefit beyond the techniques that a blind person typically develops to do things.”

Researchers were more positive about the prospects of using the implant they helped design or adopt in everyday life. Researchers R1–4, who were more closely involved with the implantees, expected the implant to be used daily or monthly for most iADLs, but also acknowledged that the implant may not be useful at all for some activities. Specifically, these researchers thought that the implant would not be used for reading, managing medication, or using personal electronic devices, but expected the implant to be most commonly used for socializing, transportation, and doing one’s laundry.

Researchers were aware of the current implants’ potential limitations in supporting reading and object recognition. When responding to question Q2-R, most researchers referred to recent R&D efforts aimed at enhancing the functionality of the current implant, citing advancements that have not yet been incorporated into the commercial version. Participant R6 opted not to comment on the implant’s effectiveness for implantees, citing a disconnect from their experiences:

“Honestly, this is a question for the users, because as an engineer, as someone who’s working on the device side, I can talk to the performance of the device but I cannot [speak to the user experience side because] I have not been involved on that side of it.”

### Implantee wishes for the next generation of implants

We further conducted a thematic analysis that revealed a total of 13 distinct themes among researcher and implantee responses (see Methods). Presented in Table 4, the theme of “irrelevance” emerges as the most frequently cited by implantees (leftmost column), signifying that they frequently find the implant unhelpful in their daily routines. This finding is somewhat anticipated for tasks like reading and identifying people, where the current implants lack the needed spatial resolution. However, it’s more unexpected for household tasks like housekeeping and meal preparation, areas where one might assume the basic vision enhancement from the implant would offer some advantage. This observation sharply contrasts with researcher expectations (center column), who had anticipated broader application of the implant across a variety of activities.

When asked to describe how an ideal future implant would assist in various iADLs (Q3-I and Q3-R), implantees mentioned a wide range of use cases (rightmost column of Table 4), spanning more themes than found in their examples of current uses.

Unsurprisingly, *vision enhancement* topped the list of desires among implantees, aligning with the core promise of bionic eye technologies. Vision enhancement here encompasses any improvement in the quality of vision the implant provides. Desired enhancements include better depth perception, as mentioned by I6, and color detection. A prevalent wish among implantees was the ability to read or recognize faces again, though there’s a shared understanding that such advancements may not be achievable with current or imminent technology. Reflecting a collective hope, all implantees resonated with I5’s desire for any improvement that would enable a shift from relying on tactile to visual cues.

The theme of *smart integration* ranked as the second most frequently mentioned by implantees. All six participants expressed a desire to see their implants work in tandem with widely-used technologies, such as barcode readers, smart glasses, text-to-speech (TTS) audio devices, and color identifiers. Participant I5 specifically noted the potential benefits of audio enhancements for tasks like meal preparation. Encouragingly, these aspirations align with ongoing research initiatives^29–33^. Researchers discussed a variety of smart integration possibilities for different iADLs, including thermal imaging for identifying hot surfaces in the kitchen, as suggested by R3, depth imaging for housekeeping, and compatibility with advanced technological aids like Microsoft’s Seeing AI and OrCam’s MyEye.

*Object identification* emerged as the third most discussed theme, encompassing the ability to discern items ranging from books to debit and credit cards in a wallet, and even bus lines. Researchers believe that current implants already have the potential to facilitate object identification for various iADLs. However, implantees view this capability more as a hope for future device enhancements.

Equally significant, the theme of *independence* stood out among implantees’ aspirations for future implants, yet it was notably absent in discussions about current technology. This discrepancy underscores a further gap between the existing capabilities of devices and the ultimate desires of implantees. Participant I5 expressed a longing for an implant that could assist in securing and maintaining employment by improving his ability to adapt and navigate the workplace.

In essence, implantees envision their implant offering benefits that surpass those provided by traditional mobility aids such as canes, guide dogs, or smartphone applications.

## Discussion

In this study, we combined quantitative and qualitative methods to explore the views of both researchers and implantees on two prominent visual prostheses, Argus II and Orion, designed for individuals experiencing vision loss. Employing both thematic and statistical analyses, we uncovered a discrepancy between researchers and implantees regarding the practical utility of these implants in daily activities. Furthermore, the study highlights the future expectations implantees have for visual implants, emphasizing the critical need for ongoing research in this field.

### Implant use falls short of researcher expectations

A key finding of our study is that the frequency and application of implant usage did not align with researchers’ anticipations. These results should be viewed in light of implantees’ existing skills in navigating blindness before implantation. Participant R2 commented on the efficiency of pre-developed strategies by implantees with employment, suggesting the implant might not enhance, and could even impede, their performance. This sentiment is encapsulated in R2’s observation:

“I suspect that [individuals] we enroll in this study, and likely those who opt for the implant later on, will have already undergone extensive training in blindness skills before getting the implant. Consequently, [getting the implant] probably won’t change how they [perform certain iADLs, as it is] easier for them to stick to the [...] method they’ve already mastered as blind individuals.”

Furthermore, all but one interviewed implantee lived with a sighted spouse or family member. This may have potentially reduced the utility of the implant in situations where spouses or family members may have aided if needed, or where preexisting assistive heuristics were established.

Yet, not all researchers fully grasp this perspective, as highlighted by R6’s admission of a disconnect from the patient experience due to his engineering role. This gap between device designers and end-users underpins a broader issue: the challenge of ensuring that clinical research aligns with the real-world needs of the blind community. This challenge is exacerbated by the fact that all interviewed researchers are sighted, complicating their ability to truly understand the lived experiences of implantees. Participant I4 felt the strongest about this:

“Researchers have no clue how it is to be blind, and do not open themselves up to opportunities [to learn about the blind community].”

We also noted a tendency among implantees to blame themselves for the device’s failures (a phenomenon initially reported by Ref.^13^). This is in stark contrast to how researchers and companies often attribute the successes of the device to its technological capabilities. For instance, Participant I2 felt his challenges were due to both the implant and his own limitations, and I5 believed his difficulty in using the implant stemmed from his low vision levels, that his “current vision level and abilities are so low that the implant doesn’t work properly.” Successes are celebrated as triumphs of technology, while failures are internalized by users as personal deficiencies.

### Implants need to compete with existing technologies

Implants have shown promise in areas like orientation and navigation, where even current aids, such as smartphone apps, fall short. Researchers acknowledge that these implants operate within a technological landscape filled with pre-existing solutions, setting a high bar for new devices to offer distinct advantages.

Therefore, visual implants might be better off focusing on fulfilling specific needs unmet by other technologies. Participant I1’s observation, “None of the [accessibility-related smartphone] apps can do what the implant can do, but the implant can’t do what any of the apps can do,” highlights the potential for implants to complement rather than compete with existing aids, leveraging their unique capabilities to fill gaps in the current assistive technology ecosystem.

### Future implants: Vision enhancement, smart integration, and independence

Our study highlights implantee wishes for future implant generations, revealing a profound desire for not only enhanced visual perception but also for greater independence. This underscores a crucial need for advancements that go beyond basic navigation aids and aim for a richer, autonomous life experience. Such feedback illuminates the complex dynamics between technological expectations, personal adaptation, and the real challenges of living with vision impairment, pointing out the stark gap in current implant discussions, which rarely touch on the crucial aspect of independence.

This technology is still in its infancy. Implantees recognize that current devices meet their expectations but look forward to substantial progress. Participant I5’s words reflect this statement:

“It met my expectations [...] We’re at Orion 1 now. Just wait till we get to Orion 15 [...] So, the faster and harder you guys work, the quicker we’ll get there.”

Integrating the perspectives and experiences of implantees into the development of future implants may be crucial to transforming this implantable device technology into a vital tool for improving quality of life. By prioritizing their experiences and needs, we can ensure that upcoming generations of implants not only push the boundaries of what is technically possible but are genuinely useful in the daily life of people who are blind.

## Figures and Tables

**Table 1.** Participant demographics for each of our interviewed implantees (I1–6). “Education” indicates the highest education level completed. “Living Situation” provides a description of other individuals that our participants currently live with, if any.

Subject	Implant	Gender	Age	Education	Employment status	Years blind	Years implanted	Residential area	Living arrangement	Mobility aid	Top accessibility aids
I1	Orion	male	25–44	High school	fully employed	11–20	1–5	city	with family	mobility cane	AI-based smartphone apps, Google maps, voiceover, JAWS
I2	Argus II	male	45–64	Master’s degree	partially employed	20+	6–10	urban	with family	mobility cane	Voiceover, many different smartphone apps
I3	Orion	male	45–64	Bachelor’s degree	not employed	11–20	1–5	urban	with sighted spouse	mobility cane	Screen reader
I4	Argus II	male	25–44	Associate’s degree	fully employed	20+	6–10	urban	with sighted spouse	guide dog	iPhone with voiceover, navigation apps, social media
I5	Argus II	male	45–64	Bachelor’s degree	not employed	20+	6–10	urban	with family	prefer not to say	Amazon Echo, Siri on phone
I6	Argus II	female	65+	Bachelor’s degree	not employed	20+	6–10	city	alone	mobility cane, guide dog	iPhone, Android phone, computer with JAWS

**Table 2.** Participant demographics for each of our interviewed researchers (R1–6). “Education” indicates the highest education level completed. “Academia/Industry” refers to the affiliation of their current work. Researchers were not asked about their living situation. None of the researchers were blind.

Subject	Implant	Gender	Age	Education	Work sector	Focus area	Years in field
R1	Argus II	female	25–44	PhD	academia	implantee-centric	5–10
R2	Argus II, Orion	male	25–44	PhD	industry, academia	implantee-centric	15–20
R3	Argus II, Orion	male	45–64	PhD	industry	both device-centric and implantee-centric	15–20
R4	Argus II	male	45–64	Bachelor’s Degree	industry, academia	implantee-centric	5–10 (with Argus, 35–40 with opth rehab)
R5	Orion	male	25–44	MD/PhD	academia	device-centric	1–5 (7th year resident, one of his projects was with Orion (trial started 2017))
R6	Argus II, Orion	male	45–64	PhD	industry	device-centric	10–15

**Table 3.** Implantee (I1–6) reports and researcher (R1–6) perception of implant use frequency for each iADL, using the scale: Daily, Weekly, Monthly, Yearly, or Never.

Subject	Meal Prep	House-keeping	Laundry	Reading	Shopping	Transportation	Finances	Medication	Personal electronic devices	Socializing	Hobbies	Employment
**I1**	Never	Yearly	Monthly	Never	Yearly	Never	Never	Never	Never	Monthly	Yearly	Never
**I2**	Never	Never	Never	Never	Never	Monthly	Never	Never	Never	Yearly	Never	Yearly
**I3**	Never	Never	Never	Never	Daily	Daily	Never	Never	Never	Daily	Weekly	Daily
**I4**	Never	Never	Never	Never	Never	Never	Never	Never	Never	Never	Never	Never
**I5**	Never	Never	Never	Never	Never	Yearly	Never	Never	Never	Yearly	Never	Never
**I6**	Never	Never	Never	Never	Never	Never	Never	Never	Never	Never	Never	Never
**R1**	Daily	Daily	Weekly	Never	Never	Daily	Never	Never	Never	Daily	Monthly	Yearly
**R2**	Never	Never	Weekly	Never	Weekly	Monthly	Never	Never	Never	Daily	Never	Weekly
**R3**	Daily	Daily	Weekly	Monthly	Weekly	Weekly	Yearly	Monthly	Monthly	Daily	Weekly	Daily
**R4**	Weekly	Weekly	Weekly	Never	Weekly	Daily	Daily	Never	Never	Daily	Weekly	Daily
**R5**	Daily	Daily	Daily	Daily	Daily	Daily	Daily	Daily	Daily	Daily	Daily	Never
**R6**	Weekly	Weekly	Weekly	Never	Weekly	Daily	Yearly	Daily	Never	Daily	Weekly	Daily

**Table 4:** Themes and definitions synthesized from an inductive thematic analysis. Numbers indicate the counts of themes: of actual implant usage as reported by implantees, of expected implant usage as reported by researchers, of implantee wishes for future visual implants.

Theme	Description	Implantee Actual Usage	Researcher Perceived Usage	Implantee Wishes
**Smart integration**	Addition of high-tech tools to implant (e.g., IR, LiDAR, audio, TTS)	0	14	20
**Accessibility aids**	Assistance from either low-tech (e.g., tactile) tools and/or people	5	17	6
**Vision enhancement**	General improvements to visual perception abilities, in cases such as reading, contrast, resolution, definition, etc	6	13	36
**Safety**	Feeling less at risk for injury (e.g., fire safety)	0	6	2
**Irrelevance**	Situations in which the implant does not help	58	25	12
**Organization**	Existing strategies around the house to keep organized (e.g., folding, keeping counters clean)	0	8	3
**Orientation**	Orienting self, mobility techniques, and avoiding obstacles	9	15	11
**Navigation**	Moving from place to place safely and accurately	8	9	12
**Independence**	Performing iADLs without external assistance	0	0	13
**Object ID**	Locating an object (not people) through computer vision or natural vision	3	16	16
**People ID**	Locating people (not objects) through computer vision or natural vision	0	7	14
**Device improvements**	Broad suggestions for future devices	0	7	7
**Enjoyment**	Use of the implant as a novel item in a non-technical way	2	2	2

## Footnotes

1
https://web.archive.org/web/20200805082212/https://www.retina-implant.de/en
